# Access to Prostate-Specific Antigen Testing and Mortality Among Men With Prostate Cancer

**DOI:** 10.1001/jamanetworkopen.2024.14582

**Published:** 2024-06-04

**Authors:** Hari S. Iyer, Benjamin V. Stone, Charlotte Roscoe, Mei-Chin Hsieh, Antoinette M. Stroup, Charles L. Wiggins, Fredrick R. Schumacher, Scarlett L. Gomez, Timothy R. Rebbeck, Quoc-Dien Trinh

**Affiliations:** 1 Section of Cancer Epidemiology and Health Outcomes, Rutgers Cancer Institute of New Jersey, New Brunswick; 2 Department of Urology and Center for Surgery and Public Health, Brigham and Women’s Hospital, Boston, Massachusetts; 3 Department of Urology, Massachusetts General Hospital, Boston; 4 Division of Population Sciences, Dana-Farber Cancer Institute, Boston, Massachusetts; 5 Department of Environmental Health, Harvard T.H. Chan School of Public Health, Boston, Massachusetts; 6 Louisiana Tumor Registry and Epidemiology Program, School of Public Health at Louisiana State University Health Sciences Center, New Orleans; 7 Department of Biostatistics and Epidemiology, Rutgers School of Public Health, Piscataway, New Jersey; 8 New Jersey State Cancer Registry, Trenton; 9 New Mexico Tumor Registry, University of New Mexico Comprehensive Cancer Center, Albuquerque; 10 Department of Population and Quantitative Health Sciences, Case Western Reserve University School of Medicine, Cleveland, Ohio; 11 Population and Cancer Prevention Program, Case Comprehensive Cancer Center, Cleveland, Ohio; 12 Department of Epidemiology and Biostatistics, University of California, San Francisco

## Abstract

**Importance:**

Prostate-specific antigen (PSA) screening for prostate cancer is controversial but may be associated with benefit for certain high-risk groups.

**Objectives:**

To evaluate associations of county-level PSA screening prevalence with prostate cancer outcomes, as well as variation by sociodemographic and clinical factors.

**Design, Setting, and Participants:**

This cohort study used data from cancer registries based in 8 US states on Hispanic, non-Hispanic Black, and non-Hispanic White men aged 40 to 99 years who received a diagnosis of prostate cancer between January 1, 2000, and December 31, 2015. Participants were followed up until death or censored after 10 years or December 31, 2018, whichever end point came first. Data were analyzed between September 2023 and January 2024.

**Exposure:**

County-level PSA screening prevalence was estimated using the Behavior Risk Factor Surveillance System survey data from 2004, 2006, 2008, 2010, and 2012 and weighted by population characteristics.

**Main Outcomes and Measures:**

Multivariable logistic, Cox proportional hazards regression, and competing risks models were fit to estimate adjusted odds ratios (AOR) and adjusted hazard ratios (AHR) for associations of county-level PSA screening prevalence at diagnosis with advanced stage (regional or distant), as well as all-cause and prostate cancer–specific survival.

**Results:**

Of 814 987 men with prostate cancer, the mean (SD) age was 67.3 (9.8) years, 7.8% were Hispanic, 12.2% were non-Hispanic Black, and 80.0% were non-Hispanic White; 17.0% had advanced disease. There were 247 570 deaths over 5 716 703 person-years of follow-up. Men in the highest compared with lowest quintile of county-level PSA screening prevalence at diagnosis had lower odds of advanced vs localized stage (AOR, 0.83; 95% CI, 0.81-0.86), lower all-cause mortality (AHR, 0.85; 95% CI, 0.84-0.86), and lower prostate cancer–specific mortality (AHR, 0.82; 95% CI, 0.79-0.84). Inverse associations between PSA screening prevalence and advanced cancer were strongest among men of Hispanic ethnicity vs other ethnicities (AOR, 0.82; 95% CI, 0.78-0.87), older vs younger men (aged ≥70 years: AOR, 0.77; 95% CI, 0.75-0.79), and those in the Northeast vs other US Census regions (AOR, 0.81; 95% CI, 0.79-0.84). Inverse associations with all-cause mortality were strongest among men of Hispanic ethnicity vs other ethnicities (AHR, 0.82; 95% CI, 0.78-0.85), younger vs older men (AHR, 0.81; 95% CI, 0.77-0.85), those with advanced vs localized disease (AHR, 0.80; 95% CI, 0.78-0.82), and those in the West vs other US Census regions (AHR, 0.89; 95% CI, 0.87-0.90).

**Conclusions and Relevance:**

This population-based cohort study of men with prostate cancer suggests that higher county-level prevalence of PSA screening was associated with lower odds of advanced disease, all-cause mortality, and prostate cancer–specific mortality. Associations varied by age, race and ethnicity, and US Census region.

## Key Points

**Question:**

Is county-level prevalence of prostate-specific antigen (PSA) screening associated with lower mortality among men with prostate cancer?

**Findings:**

In this population-based multistate cohort study of 814 987 men with prostate cancer followed up for up to 10 years, higher prevalence of county-level PSA screening in the 2 years prior to diagnosis was associated with lower odds of advanced stage cancer, all-cause mortality, and prostate cancer–specific mortality. Inverse associations with overall mortality were strongest among men who were 70 years or older, were Hispanic, had regional or distant disease at diagnosis, and resided in the West US Census region.

**Meaning:**

This study suggests that men residing in counties with higher PSA screening prevalence prior to diagnosis had lower all-cause and prostate cancer-specific mortality; associations varied by sociodemographic and geographic factors, suggesting that targeted screening and linkage to treatment could improve prostate cancer survival.

## Teaser

This cohort study evaluates associations of county-level prostate-specific antigen (PSA) screening prevalence with prostate cancer outcomes, including mortality, as well as variation by sociodemographic and clinical factors.

## Introduction

Prostate cancer is a leading cause of cancer-related morbidity and mortality, accounting for 288 300 new cases and 34 700 deaths each year.^1^ Few consistent modifiable risk factors for prostate cancer have been identified, limiting the scope for primary prevention.^2^ Use of prostate-specific antigen (PSA) screening is widespread in the US but controversial. In 2012, based on limited evidence of survival benefits from clinical trials,^3,4^ the United States Preventive Services Task Force (USPSTF) issued recommendations advising against use of PSA screening.^5^ In the years that followed, screening decreased,^6,7^ and incidence of de novo metastatic prostate cancer increased, with early reports suggesting reversal of gains in prostate cancer–specific mortality reduction.^8,9,10,11^ These concerning trends led the USPSTF in 2018 to slightly revise its recommendations, allowing for shared decision-making about whether or not to receive PSA screening.^12,13^

For those at higher risk of aggressive prostate cancer, the benefits from earlier detection may outweigh the harms of overtreatment.^14,15^ Emerging screening methods for prostate cancer that may yield more favorable harm-benefit trade-offs include screening midlife PSA levels,^16^ emerging molecular subtypes,^17^ genetic risk scores,^18^ and the development of race-conscious guidelines for Black and African American men.^19^ Although individual-level data on screening and survival are rarely available, area-level estimates of screening allow for the investigation of how access to PSA screening may be associated with prostate cancer outcomes in diverse populations.

Although prior studies to evaluate trends in changing screening patterns and mortality have been conducted using the Surveillance, Epidemiology, and End Results (SEER) database and nationwide surveys of health care behaviors, most used ecological designs or modeling approaches.^14,20,21^ To our knowledge, few have incorporated subcounty-level measures of socioeconomic status (SES), segregation, and environmental factors with individual-level case data to evaluate associations of screening with mortality.^22^ Examining associations of screening with mortality in a population-based study with more precise geospatial measures of social and built environments may offer more reliable estimates of potential associations of targeted screening with prostate cancer outcomes.^23,24,25^ In addition, using databases with wide geographic coverage could identify population subgroups of individuals for whom screening could offer greater benefits.^26^

Using a novel multistate population-based registry cohort of men with a diagnosis of prostate cancer, we investigated whether county-level prevalence of PSA screening was associated with lower stage at diagnosis and lower mortality. We also examined whether associations varied by sociodemographic and geographic characteristics.

## Methods

### Study Population and Design

We used data from the Multilevel Epidemiologic Tumor Registry for Oncology (METRO), a registry-based cohort study of men with a diagnosis of prostate cancer from the California, Louisiana, Massachusetts, New Jersey, New Mexico, Ohio, Pennsylvania, and Seattle–Puget Sound cancer registries. These registries were chosen to capture a geographic and sociodemographic diversity of experiences of men with a diagnosis of prostate cancer in the US. Registries contributing data to the METRO prostate cancer cohort are all part of the North American Association of Central Cancer Registries (NAACCR)^27^ and National Program of Cancer Registries (NPCR). A subset of registries is part of the SEER program (California, Louisiana, Massachusetts, New Jersey, New Mexico, and Seattle–Puget Sound).^28^ The NAACCR, the NPCR, and the SEER database require standardized reporting and quality checks to ensure the validity of the information collected and to allow for harmonization of clinical and sociodemographic database elements for surveillance. Deaths are updated at minimum annually through verification of state and national death records, including for deaths of patients who moved out of state. Address-level geospatial data linkages are described in more detail in the eMethods in Supplement 1. The institutional review boards of the Dana-Farber Cancer Institute and Rutgers University, The State University of New Jersey, approved this study and determined that, because existing data sources were used, no written consent was required for participation in the study. Access to cancer registry data additionally required state institutional review board approval for California, Massachusetts, and New Jersey. This study followed the Strengthening the Reporting of Observational Studies in Epidemiology (STROBE) reporting guideline.

Hispanic, non-Hispanic Black, and non-Hispanic White men aged 40 to 99 years at diagnosis with a first primary prostate cancer between January 1, 2000, and December 31, 2015, were included in the study. These racial and ethnic groups were chosen because prostate cancer incidence rates are highest in these groups,^1,29^ and the magnitude and persistence of the Black-White racial disparity in prostate cancer incidence and mortality has been an ongoing public health challenge.^30,31,32,33,34^ Race and ethnicity were assigned by cancer registrars based on information recorded in medical records, vital records, and sometimes imputation algorithms based on surname and country of origin.^35^ Men were followed up until death from any cause, December 31, 2018, or up to 10 years from diagnosis, whichever came first. We excluded men who were missing data on county-level PSA screening or who were missing covariate data (n = 11 334), for a final analytic sample size of 814 987 (eFigure 1 in Supplement 1).

### County-Level Prevalence of PSA Testing

County-level estimates of PSA testing, which reflect availability of and physical accessibility to screening, were estimated using a previously validated approach for extrapolating prevalence estimates of health behaviors from the Behavioral Risk Factor Surveillance System (BRFSS) to the total US population using a multilevel mixed-effects model and poststratification weights.^36,37,38,39^ In brief, the BRFSS is a telephone-based survey conducted annually among US adults to monitor state and national trends in health-related behaviors and use of preventive services. Questions about PSA screening (whether self-identified male respondents ever received a PSA test, and how long ago the test was conducted) were asked in even-numbered years. Data on population sociodemographic characteristics (age, race and ethnicity, and poverty) were obtained from the 2000 and 2010 decennial US Censuses and the 2010 American Community Survey and linked to participant’s year of diagnosis. These population-level data were used to calculate weighted population-based screening prevalence for each year in each county.

We obtained BRFSS data for the years 2004, 2006, 2008, 2010, and 2012, for which data on use of PSA screening were collected from men across all US states. We restricted the BRFSS sample to men between 40 and 79 years of age with information on county of residence who did not report a history of prostate cancer. Further details regarding the methods were described previously.^40^ For our primary analysis, we linked county-level prevalence of PSA testing during the 2-year period prior to year of METRO participant’s diagnosis. For those diagnosed before 2004, we linked county-level prevalence of PSA testing in 2004, and for those diagnosed after 2012, we linked prevalence data from 2012.

### Outcomes

Our primary goal was to estimate associations of county-level PSA screening with survival. We hypothesized that men living in counties with a higher prevalence of PSA screening would be more likely to be screened, leading to a lower disease stage at diagnosis and longer survival. Our primary end points were all-cause mortality and prostate cancer–specific mortality. Our secondary end point was SEER Summary Stage^41^ at diagnosis (localized vs regional or distant disease).

Mortality was ascertained by registries through linkages with state death certificates and the National Death Index, enabling capture of deaths that occurred out of state. Prostate cancer–specific mortality was assessed using the *International Classification of Diseases, Ninth Revision* (code 185) and the *International Statistical Classification of Diseases and Related Health Problems, Tenth Revision* (code C61).^42^

### Statistical Analysis

Data were analyzed between September 2023 and January 2024. We summarized associations of sociodemographic and neighborhood factors with county-level prostate cancer screening at diagnosis. For all-cause mortality, we fit 3 Cox proportional hazards regression models that were sequentially adjusted for variables. Model 1 (minimal) was adjusted for age (5-year categories) and diagnosis year (categorical: 2000-2004, 2005-2009, and 2010-2015). Model 2 (full) was adjusted for race and ethnicity (categorical: Hispanic, non-Hispanic Black, and non-Hispanic White); neighborhood SES index, calculated using a *z* score–scaled summary measure^43^ (quintiles); racialized income Index of Concentration at the Extremes, a measure of segregation^44^ (quintiles); Normalized Difference Vegetation Index, a satellite-derived measure of green space^45,46^ (continuous); modeled ambient particulate matter within 2.5 µm in diameter (quadratic)^47^; population density (people per square mile; quadratic); and state (categorical). Model 3 (health system) was adjusted for urologist density per 1000 (continuous), physician density per 1000 (continuous), and proximity to closest cancer hospital by public transportation travel zone generated using Google Maps (<30 minutes, 30-60 minutes, and >60 minutes). Clinician density was obtained from the Area Health Resource File 2012. Neighborhood covariates were selected based on prior evidence for associations with prostate cancer outcomes.^22,25,48,49,50,51^ For mortality models, we considered further adjustment by receipt of surgery, receipt of radiotherapy, and stage at diagnosis, but including these covariates did not alter effect estimates. Models for prostate cancer–specific mortality were fit with the same sets of covariates using inverse probability weights for competing risks^52^ (eMethods in Supplement 1). We fit logistic regression models for stage at diagnosis (binary: localized vs regional or distant [advanced stage]) using the same sets of covariates for models 1, 2, and 3.

We evaluated effect modification of associations of county-level PSA screening with advanced stage and all-cause mortality using likelihood ratio tests for multiplicative interaction and adjusting for covariates specified in model 2. We assessed race and ethnicity, age, neighborhood SES, and US Census region as modifiers. For all-cause mortality models, we additionally included disease stage as a modifier. We repeated analyses using different time windows for county-level screening (baseline [2004], cumulative updated average from 2004 through year of diagnosis) as sensitivity analyses. Race and ethnicity–specific county-level screening estimates and county-level mammography were evaluated as additional sensitivity analyses (eMethods in Supplement 1). Details about geospatial data sources and exposure windows are described in more detail in the eMethods in Supplement 1. All analyses were performed using R, version 4.2.2 (R Project for Statistical Computing). All *P* values were from 2-sided hypothesis tests, and results were deemed statistically significant at *P* < .05.

## Results

This study included 814 987 men with prostate cancer (mean [SD] age, 67.3 [9.8] years); 17.0% had advanced disease (eTable 1 in Supplement 1). A total of 7.8% were Hispanic, 12.2% were non-Hispanic Black, and 80.0% were non-Hispanic White. Compared with those in the lowest quintile, men residing in counties in the highest quintile of prevalence of PSA screening were more likely to be non-Hispanic White (87.4% vs 73.4%), more likely to be covered by Medicare (46.6% vs 39.0%), less likely to be diagnosed from 2010 to 2015 (34.5% vs 50.9%), and more likely to reside in low SES neighborhoods (23.0% vs 12.6%). The prevalence of localized disease stage at diagnosis was higher among men in counties with the highest quintile compared with lowest quintile of PSA screening (85.3% vs 80.2%). There were 247 570 deaths over 5 716 703 person-years of follow-up.

County-level screening was associated with lower all-cause mortality among men with prostate cancer (eFigure 2 in Supplement 1). Men in higher compared with lower quintiles of county-level PSA had lower all-cause mortality. Results were similar when compared with analyses using baseline (2004) and cumulative updated average county-level PSA screening.

In fully adjusted models, men residing in counties with the highest prevalence of PSA testing had 17% lower odds of advanced stage diagnosis (quintile 5 vs quintile 1: adjusted odds ratio [AOR], 0.83; 95% CI, 0.81-0.86; *P* < .001 for trend) (Table 1). In sensitivity analysis, men living in the highest compared with lowest quintile of county-level PSA screening at baseline (AOR, 0.87; 95% CI, 0.85-0.90; *P* < .001 for trend) and using cumulative updated average county-level screening through diagnosis (AOR, 0.87; 95% CI, 0.85-0.90; *P* < .001 for trend) had statistically significantly lower odds of advanced stage disease at diagnosis.

**Table 1.** Adjusted Odds Ratios for Association Between County-Level PSA Testing Prevalence and Stage at Diagnosis Among 583 597 Men With a Diagnosis of Prostate Cancer

County-level PSA prevalence^a^	Adjusted odds ratio (95% CI)						*P* value for trend
	Continuous (10%)	Quintile 1	Quintile 2	Quintile 3	Quintile 4	Quintile 5	
**Diagnosis**							
Advanced vs localized disease, No.	NA	28 646/115 820	20 624/97 249	18 220/91 969	17 431/96 050	14 378/83 210	NA
Age^b^	0.78 (0.77-0.79)	1.00 [Reference]	0.89 (0.87-0.90)	0.84 (0.82-0.86)	0.77 (0.75-0.79)	0.71 (0.70-0.73)	<.001
Full^c^	0.86 (0.85-0.88)	1.00 [Reference]	0.94 (0.92-0.96)	0.87 (0.85-0.89)	0.85 (0.83-0.87)	0.83 (0.81-0.86)	<.001
Full plus HSR^d^	0.86 (0.85-0.88)	1.00 [Reference]	0.95 (0.93-0.97)	0.89 (0.87-0.91)	0.85 (0.83-0.87)	0.84 (0.82-0.87)	<.001
**Baseline (2004)**							
Advanced vs localized disease, No.	NA	25 153/104 993	19 986/97 266	17 895/94 033	18 186/91 356	18 079/96 650	NA
Age^b^	0.83 (0.82-0.85)	1.00 [Reference]	0.85 (0.83-0.86)	0.77 (0.75-0.79)	0.82 (0.80-0.84)	0.76 (0.75-0.78)	<.001
Full^c^	0.93 (0.91-0.95)	1.00 [Reference]	0.95 (0.92-0.97)	0.82 (0.80-0.84)	0.89 (0.86-0.91)	0.87 (0.85-0.90)	<.001
Full plus HSR^d^	0.96 (0.94-0.99)	1.00 [Reference]	0.99 (0.96-1.02)	0.88 (0.85-0.91)	0.91 (0.88-0.94)	0.93 (0.90-0.97)	<.001
**Cumulative updated average**							
Advanced vs localized disease, No.	NA	25 720/11 0170	19 878/91 397	19 465/95 992	18 792/98 947	15 444/87 792	NA
Age^b^	0.77 (0.75-0.78)	1.00 [Reference]	0.90 (0.88-0.92)	0.83 (0.81-0.85)	0.78 (0.76-0.80)	0.73 (0.72-0.75)	<.001
Full^c^	0.90 (0.87-0.92)	1.00 [Reference]	0.95 (0.93-0.97)	0.89 (0.87-0.91)	0.88 (0.86-0.90)	0.87 (0.85-0.90)	<.001
Full plus HSR^d^	0.92 (0.89-0.95)	1.00 [Reference]	0.97 (0.95-1.00)	0.93 (0.90-0.95)	0.90 (0.88-0.93)	0.92 (0.89-0.95)	<.001

Abbreviations: HSR, Health Services Resources; NA, not applicable; PSA, prostate-specific antigen.

^a^ Time windows: diagnosis = 2 years prior to diagnosis; baseline = earliest estimate (2004); cumulative updated average = average over all years prior to diagnosis.

^b^ Model adjusted for age and diagnosis year.

^c^ Model further adjusted for race and ethnicity, neighborhood socioeconomic status, racialized income Index of Concentration at the Extremes, greenness, air pollution (particulate matter within 2.5 µm in diameter), population density, and state.

^d^ Model further adjusted for urologist density, radiation oncologist density, physician density, and proximity to cancer care facility.

Men residing in counties with the highest prevalence of PSA testing had 14% lower all-cause mortality (quintile 5 vs quintile 1: adjusted hazard ratio [AHR], 0.85; 95% CI, 0.84-0.86; *P* < .001 for trend) (Table 2). Men living in the highest compared with lowest quintile of county-level PSA screening at baseline (AHR, 0.97; 95% CI, 0.96-0.99; *P* < .001 for trend) and using cumulative updated average county-level screening through diagnosis (AHR, 0.91; 95% CI, 0.89-0.92; *P* < .001 for trend) had statistically significantly lower all-cause mortality, although associations were attenuated.

**Table 2.** Adjusted Hazard Ratios for Association Between County-Level PSA Testing Prevalence and All-Cause Mortality Among 814 987 Men With a Diagnosis of Prostate Cancer

County-level PSA prevalence^a^	Adjusted hazard ratio (95% CI)						*P* value for trend
	Continuous (10%)	Quintile 1	Quintile 2	Quintile 3	Quintile 4	Quintile 5	
**Diagnosis**							
Deaths/person-years	NA	47 470/1 034 252	43 862/1 073 696	54 544/1 205 249	52 998/1 202 205	48 696/1 201 301	NA
Age^b^	0.88 (0.88-0.89)	1.00 [Reference]	0.92 (0.90-0.93)	0.97 (0.95-0.98)	0.93 (0.92-0.94)	0.85 (0.84-0.86)	<.001
Full^c^	0.86 (0.85-0.87)	1.00 [Reference]	0.94 (0.93-0.95)	0.94 (0.93-0.95)	0.88 (0.86-0.89)	0.85 (0.84-0.86)	<.001
Full plus HSR^d^	0.86 (0.85-0.87)	1.00 [Reference]	0.94 (0.92-0.95)	0.93 (0.92-0.95)	0.87 (0.86-0.88)	0.84 (0.83-0.86)	<.001
**Baseline (2004)**							
Deaths/person-years	NA	52 048/1 194 640	46 494/1 177 648	48 454/1 051 264	51 395/1 120 334	49 179/1 172 818	NA
Age^b^	0.96 (0.95-0.96)	1.00 [Reference]	0.92 (0.91-0.93)	1.01 (1.00-1.03)	1.02 (1.00-1.03)	0.91 (0.90-0.93)	<.001
Full^c^	0.96 (0.95-0.98)	1.00 [Reference]	0.99 (0.98-1.01)	1.00 (0.98-1.01)	0.99 (0.98-1.00)	0.97 (0.96-0.99)	<.001
Full plus HSR^d^	0.96 (0.95-0.97)	1.00 [Reference]	1.00 (0.99-1.02)	1.00 (0.99-1.02)	1.00 (0.98-1.01)	0.98 (0.96-0.99)	.002
**Cumulative updated average**							
Deaths/person-years	NA	52 274/1 183 850	46 388/1 111 281	50 228/1 106 527	48 670/1 114 405	50 010/1 200 640	NA
Age^b^	0.93 (0.92-0.93)	1.00 [Reference]	0.96 (0.94-0.97)	1.00 (0.99-1.01)	0.96 (0.94-0.97)	0.89 (0.88-0.91)	<.001
Full^c^	0.92 (0.91-0.93)	1.00 [Reference]	0.99 (0.98-1.00)	0.98 (0.97-1.00)	0.93 (0.91-0.94)	0.91 (0.89-0.92)	<.001
Full plus HSR^d^	0.91 (0.90-0.93)	1.00 [Reference]	0.99 (0.98-1.01)	0.98 (0.97-1.00)	0.92 (0.91-0.94)	0.90 (0.89-0.92)	<.001

Abbreviations: HSR, Health Services Resources; NA, not applicable; PSA, prostate-specific antigen.

^a^ Time windows: diagnosis = 2 years prior to diagnosis; baseline = earliest estimate (2004); cumulative updated average = average over all years prior to diagnosis.

^b^ Model adjusted for age and diagnosis year.

^c^ Model further adjusted for race and ethnicity, neighborhood socioeconomic status, racialized income Index of Concentration at the Extremes, greenness, air pollution (particulate matter within 2.5 µm in diameter), population density, and state.

^d^ Model further adjusted for urologist density, radiation oncologist density, physician density, and proximity to cancer care facility.

Men residing in counties with the highest prevalence of PSA testing had 17% lower prostate cancer–specific mortality (quintile 5 vs quintle 1: AHR, 0.82; 95% CI, 0.79-0.84; *P* < .001 for trend) (Table 3). In sensitivity analysis, men living in the highest compared with lowest quintile of county-level PSA screening at baseline (AHR, 0.94; 95% CI, 0.91-0.97; *P* < .001 for trend) and using cumulative updated average county-level screening through diagnosis (AHR, 86; 95% CI, 0.84-0.89; *P* < .001 for trend) had statistically significantly lower prostate cancer–specific mortality, although associations were attenuated.

**Table 3.** Adjusted Hazard Ratios for Association Between County-Level PSA Testing Prevalence and Prostate Cancer–Specific Mortality Among 814 987 Men With a Diagnosisis of Prostate Cancer

County-level PSA prevalence^a^	Adjusted hazard ratio (95% CI)						*P* value for trend
	Continuous (10%)	Quintile 1	Quintile 2	Quintile 3	Quintile 4	Quintile 5	
**Diagnosis**							
Deaths/person-years	NA	16 209/1 034 252	14 678/1 073 696	15 015/1 205 249	14 816/1 202 205	12 760/1 201 301	NA
Age^b^	0.81 (0.80-0.83)	1.00 [Reference]	0.95 (0.92-0.97)	0.91 (0.89-0.94)	0.89 (0.87-0.91)	0.77 (0.75-0.79)	<.001
Full^c^	0.83 (0.81-0.85)	1.00 [Reference]	0.94 (0.92-0.97)	0.93 (0.90-0.95)	0.85 (0.83-0.87)	0.82 (0.79-0.84)	<.001
Full plus HSR^d^	0.83 (0.81-0.84)	1.00 [Reference]	0.94 (0.92-0.96)	0.92 (0.90-0.95)	0.85 (0.82-0.87)	0.81 (0.79-0.84)	<.001
**Baseline (2004)**							
Deaths/person-years	NA	16 121/1 194 640	15 858/1 177 648	13 625/1 051 264	14 861/1 120 334	13 013/1 172 818	NA
Age^b^	0.88 (0.87-0.90)	1.00 [Reference]	0.98 (0.96-1.00)	0.96 (0.94-0.99)	0.99 (0.97-1.02)	0.82 (0.80-0.84)	<.001
Full^c^	0.93 (0.91-0.95)	1.00 [Reference]	1.02 (0.99-1.05)	0.98 (0.96-1.01)	0.98 (0.96-1.01)	0.94 (0.91-0.97)	<.001
Full plus HSR^d^	0.93 (0.90-0.95)	1.00 [Reference]	1.02 (1.00-1.05)	0.98 (0.95-1.01)	0.99 (0.96-1.02)	0.94 (0.91-0.97)	<.001
**Cumulative updated average**							
Deaths/person-years	NA	168 11/1 183 850	15 269/1 111 281	14 581/1 106 527	13 845/1 114 405	12 972/1 200 640	NA
Age^b^	0.84 (0.82-0.85)	1.00 [Reference]	0.97 (0.95-0.99)	0.93 (0.91-0.95)	0.89 (0.87-0.91)	0.79 (0.77-0.81)	<.001
Full^c^	0.87 (0.85-0.90)	1.00 [Reference]	1.00 (0.97-1.02)	0.96 (0.93-0.98)	0.90 (0.87-0.93)	0.86 (0.84-0.89)	<.001
Full plus HSR^d^	0.87 (0.84-0.89)	1.00 [Reference]	1.00 (0.97-1.02)	0.95 (0.93-0.98)	0.89 (0.87-0.92)	0.86 (0.83-0.89)	<.001

Abbreviations: HSR, Health Services Resources; NA, not applicable; PSA, prostate-specific antigen.

^a^ Models were fit using stabilized inverse probability weights for competing event of non-prostate cancer–specific deaths. Time windows: diagnosis = 2 years prior to diagnosis, baseline = earliest estimate (2004), cumulative updated average = average over all years prior to diagnosis.

^b^ Model adjusted for age and diagnosis year.

^c^ Model further adjusted for race and ethnicity, neighborhood socioeconomic status, racialized income Index of Concentration at the Extremes, greenness, air pollution (particulate matter within 2.5 µm in diameter), population density, and state.

^d^ Model further adjusted for urologist density, radiation oncologist density, physician density, and proximity to cancer care facility.

We evaluated sociodemographic and geographic modifiers for the associations of county-level prevalence of PSA screening and advanced disease stage at diagnosis (Table 4). The inverse association between county-level PSA screening at diagnosis and advanced stage was weakest for non-Hispanic Black men (AOR, 0.93; 95% CI, 0.88-0.97) and strongest for Hispanic men (AOR, 0.82; 95% CI, 0.78-0.87; *P* = .002 for interaction). Inverse associations were strongest for older men (aged ≥70 years: AOR, 0.77; 95% CI, 0.75-0.79; *P* < .001 for interaction) and for those living in the Northeast Census region (AOR, 0.81; 95% CI, 0.79-0.84; *P* < .001 for interaction). Associations were similar for baseline and cumulative updated average county-level PSA screening.

**Table 4.** Adjusted ORs for Association Between County-Level PSA Testing Prevalence and Stage (Distant or Regional vs Localized) Among 583 597 Men With a Diagnosis of Prostate Cancer

County-level PSA (10%)	Adjusted OR (95% CI) at diagnosis^a^	*P* value for interaction	Adjusted OR (95% CI) at baseline (2004)^a^	*P* value for interaction	Adjusted OR (95% CI) for cumulative updated average^a^	*P* value for interaction
Race and ethnicity						
Hispanic	0.82 (0.78-0.87)	.002	0.82 (0.77-0.88)	<.001	0.77 (0.71-0.83)	<.001
Non-Hispanic Black	0.93 (0.88-0.97)		0.90 (0.86-0.95)		0.89 (0.84-0.95)	
Non-Hispanic White	0.86 (0.84-0.88)		0.95 (0.92-0.97)		0.91 (0.88-0.94)	
Age, y						
40-54	0.99 (0.94-1.03)	<.001	0.97 (0.93-1.02)	.02	0.95 (0.90-1.01)	.02
55-69	0.91 (0.89-0.93)		0.94 (0.91-0.97)		0.91 (0.88-0.94)	
≥70	0.77 (0.75-0.79)		0.91 (0.88-0.94)		0.87 (0.84-0.91)	
nSES						
Quintile 1 (low)	0.87 (0.84-0.90)	.19	0.90 (0.86-0.94)	.63	0.88 (0.84-0.93)	.40
Quintile 2	0.84 (0.81-0.87)		0.93 (0.89-0.97)		0.89 (0.85-0.94)	
Quintile 3	0.86 (0.83-0.89)		0.93 (0.89-0.96)		0.89 (0.85-0.93)	
Quintile 4	0.86 (0.83-0.89)		0.94 (0.90-0.98)		0.90 (0.86-0.94)	
Quintile 5 (high)	0.89 (0.86-0.92)		0.94 (0.90-0.97)		0.93 (0.89-0.97)	
US Census region						
Northeast	0.81 (0.79-0.84)	<.001	0.84 (0.81-0.88)	<.001	0.81 (0.78-0.85)	<.001
Midwest	0.90 (0.85-0.96)		1.07 (1.00-1.14)		1.21 (1.13-1.30)	
South	0.86 (0.79-0.94)		0.79 (0.72-0.88)		0.77 (0.66-0.89)	
West	0.88 (0.86-0.90)		0.92 (0.90-0.94)		0.89 (0.87-0.91)	

Abbrevations: nSES, neighborhood socioeconomic status; OR, odds ratio; PSA, prostate-specific antigen.

^a^ Models adjusted for age, diagnosis year, race, nSES, racialized income Index of Concentration at the Extremes, greenness, air pollution (particulate matter within 2.5 µm in diameter), population density, and state. Time windows: diagnosis = 2 years prior to diagnosis; baseline = earliest estimate (2004); cumulative updated average = average over all years prior to diagnosis.

Associations of county-level PSA screening and all-cause mortality also varied by sociodemographic factors (Table 5). Hispanic men residing in counties with a higher prevalence of PSA screening had lower all-cause mortality (AHR per 10% increase, 0.82; 95% CI, 0.78-0.85; *P* = .009 for interaction). Inverse associations between higher prevalence of county-level PSA screening and all-cause mortality were strongest among younger men (aged 40-54 years: AHR, 0.81; 95% CI, 0.77-0.85; *P* < .001 for interaction), those with advanced stage disease (AHR, 0.80; 95% CI, 0.78-0.82; *P* <. 001 for interaction), and those residing in the West Census region (AHR, 0.89; 95% CI, 0.87-0.90; *P* < .001 for interaction). Effect modification results varied by timing of PSA exposure, with evidence of variation by neighborhood SES and more limited evidence of effect modification by race and ethnicity and US Census region observed for baseline and cumulative updated average models.

**Table 5.** Adjusted HRs for Association Between County-Level PSA and All-Cause Mortality Among 814 987 Men With a Diagnosis of Prostate Cancer

County-level PSA (10%)	Adjusted HR (95% CI) at diagnosis^a^	*P* value for interaction	Adjusted HR (95% CI) at baseline (2004)^a^	*P* value for interaction	Adjusted HR (95% CI) for cumulative updated average^a^	*P* value for interaction
Race and ethnicity						
Hispanic	0.82 (0.78-0.85)	.009	1.03 (0.98-1.07)	.005	0.94 (0.89-0.98)	.48
Non-Hispanic Black	0.88 (0.85-0.90)		0.98 (0.95-1.00)		0.93 (0.90-0.96)	
Non-Hispanic White	0.86 (0.85-0.87)		0.96 (0.95-0.97)		0.92 (0.90-0.93)	
Age, y						
40-54	0.81 (0.77-0.85)	<.001	0.94 (0.90-0.99)	.12	0.89 (0.84-0.94)	.03
55-69	0.85 (0.84-0.86)		0.97 (0.95-0.98)		0.92 (0.90-0.94)	
≥70	0.88 (0.86-0.89)		0.98 (0.97-1.00)		0.94 (0.93-0.95)	
nSES						
Quintile 1 (low)	0.87 (0.85-0.88)	.21	0.92 (0.90-0.94)	<.001	0.90 (0.88-0.92)	.03
Quintile 2	0.86 (0.85-0.88)		0.97 (0.95-0.99)		0.93 (0.91-0.95)	
Quintile 3	0.87 (0.86-0.89)		0.99 (0.97-1.01)		0.94 (0.92-0.97)	
Quintile 4	0.85 (0.84-0.87)		0.97 (0.95-0.99)		0.92 (0.90-0.95)	
Quintile 5 (high)	0.85 (0.83-0.87)		0.99 (0.97-1.02)		0.92 (0.90-0.95)	
Stage^b^						
Localized	0.87 (0.86-0.88)	<.001	0.97 (0.95-0.99)	<.001	0.92 (0.90-0.93)	<.001
Regional or distant	0.80 (0.78-0.82)		1.01 (0.99-1.04)		0.94 (0.92-0.97)	
US Census region^c^						
Northeast	0.90 (0.88-0.92)	<.001	0.98 (0.96-1.00)	<.001	0.90 (0.88-0.92)	<.001
Midwest	0.94 (0.92-0.96)		0.99 (0.97-1.01)		1.03 (1.01-1.05)	
South	0.96 (0.92-1.01)		0.93 (0.88-0.98)		0.97 (0.90-1.03)	
West	0.89 (0.87-0.90)		1.02 (1.01-1.03)		1.00 (0.98-1.01)	

Abbreviations: HR, hazard ratio; nSES, neighborhood socioeconomic status; PSA, prostate-specific antigen.

^a^ Models adjusted for age, diagnosis year, race, nSES, racialized income Index of Concentration at the Extremes, greenness, air pollution (particulate matter within 2.5 µm in diameter), population density, and state. Time windows: diagnosis = 2 years prior to diagnosis; baseline = earliest estimate (2004); cumulative updated average = average over all years prior to diagnosis.

^b^ Restricted to sample with complete stage information (n = 583 601).

^c^ Models not adjusted for state.

In sensitivity analyses using race and ethnicity–specific estimates of PSA screening within racial and ethnic groups, there was no variation in associations with advanced stage by race and ethnicity (eTable 2 in Supplement 1). Higher prevalence of race and ethnicity–specific screening was more strongly associated with lower all-cause and prostate cancer–specific mortality among non-Hispanic White compared with non-Hispanic Black individuals, although 95% CIs overlapped. Compared with analyses with county-level PSA screening, associations of county-level mammography screening in years prior to diagnosis were of similar magnitude and statistically significantly associated with lower advanced disease stage at diagnosis compared with county-level PSA screening, but they were less strongly associated with all-cause and prostate cancer–specific mortality (eTable 2 in Supplement 1).

## Discussion

In this multistate population-based cohort study across 8 cancer registries, residence in counties with a higher prevalence of PSA screening was associated with lower odds of advanced disease stage, lower all-cause mortality, and lower prostate cancer–specific mortality. Associations varied by sociodemographic and geographic factors, with stronger associations observed in different age groups, Hispanic men, and US Census regions.

Current guidelines for PSA screening are based largely on evidence from randomized clinical trials. This evidence is mixed,^53^ with the strongest clinical trial evidence coming from the Göteborg trial and the European Randomized Study of Screening for Prostate Cancer (ERSPC), which found lower mortality among those who received screening over 10 to 16 years.^54,55^ However, many other clinical trials, including the Prostate, Lung, Colorectal, and Ovarian (PLCO) trial in the US and the Cluster Randomized Trial of PSA Testing for Prostate Cancer (CAP) in the UK, both of which tested low-intensity screening, found no survival benefits.^3,56,57^ These trials were not powered to assess harm-benefit trade-offs among men at high risk, such as those with genetic susceptibility or Black men.^19^ Moreover, clinical trials did not capture the barriers to access that patients face when navigating care in the broader health system,^58^ which may contribute to racial and ethnic disparities.^32,59,60^

Our study, focused on county-level PSA screening as a proxy for access to screening, aimed to provide evidence regarding the possible benefits associated with population-based screening in high-risk sociodemographic groups. Evaluations using the SEER databases have suggested that widespread use of PSA testing in the late 1980s and early 1990s contributed to overdiagnosis of indolent tumors and unnecessary treatment.^21,61,62^ However, these evaluations also suggest that decreasing trends in mortality over the past 20 years are associated in part with earlier detection of metastatic tumors of as much as 45% to 70%.^21^ Members of our team previously conducted an ecological county-level study using SEER data, finding that a higher prevalence of PSA screening was associated with a lower incidence of metastatic prostate cancer and lower prostate cancer–specific survival, with estimates similar to results presented here.^40^ Research in the Veterans Health Administration found that men seeking care in facilities with higher PSA screening rates had roughly 10% lower rates of metastatic prostate cancer at presentation associated with a 10% increase in screening rate,^63^ comparable with our estimates. Additional analyses in the Veterans Health Administration suggest that Black men with greater frequency of screening may experience a lower risk of prostate cancer–specific mortality.^64^ Our findings suggest that possible influences of PSA screening resources and accessibility vary across different geographic and sociodemographic groups, independent of neighborhood social context and environmental factors. Further research using databases that capture health care access barriers along the care continuum and more detailed treatment information may explain these patterns.

### Strengths and Limitations

This study has some strengths, including its size, capture of address-level neighborhood contextual factors and environmental confounders, and the sociodemographic and geographic diversity of the study population. This study also has some limitations. We lacked measures of participants’ actual screening history, and our exposure is better understood as a reflection of the availability of PSA screening resources in their county of residence. In particular, we could not examine subcounty variation in race and ethnicity–specific use of PSA screening. Prevalence of PSA screening is estimated from self-report, which may be susceptible to recall bias and social desirability bias. However, by acquiring exposure and outcome data from different databases, we reduce the risk of differential measurement error. Confounding by unmeasured comorbidities and lifestyle factors is possible, although we adjusted for important neighborhood socioeconomic and environmental factors that could partly account for this. Cause of death ascertainment can vary by geographic and clinician-level factors, and a participant’s death may be incorrectly attributed to their cancer diagnosis instead of the true underlying cause of death.^65,66^ We assume that after adjustment for covariates, this bias is nondifferential. We lacked precise measures of tumor aggressiveness (Gleason score and molecular markers) and so could not formally evaluate mediation by tumor severity.

## Conclusions

In this cohort study, we found that men with prostate cancer residing in US counties with a higher prevalence of PSA screening experienced lower odds of advanced stage disease and lower rates of all-cause and prostate cancer–specific mortality. Associations varied based on sociodemographic and geographic characteristics, supporting calls for tailored screening recommendations in population subgroups with a higher risk of aggressive disease. Future studies should incorporate information on care-seeking behaviors.

## Notes

Supplement 1. **eMethods.** Geospatial Covariates and Time-Varying Exposure
eReferences.
**eFigure 1.** CONSORT Diagram for METRO Prostate Cancer Cohort Study of County-Level Prostate-Specific Antigen Testing and Mortality **eFigure 2.** Kaplan-Meier Survival Curves for County-Level PSA at (A) Baseline, (B) Diagnosis, (C) Cumulative Updated Average in METRO **eTable 1.** Descriptive Characteristics of the Multilevel Tumor Registry for Evaluation of Oncology (METRO) Cohort of Men With Prostate Cancer Stratified by County-Level Prevalence of Prostate Specific Antigen Testing at Diagnosis **eTable 2.** Sensitivity Analyses for Associations of County-Level Prevalence of Screening With Advanced Stage, All-Cause Mortality and Prostate Cancer-Specific Mortality Stratified by Race (Prostate-Specific Antigen), and With Mammography as a Negative Control

Supplement 2.
Data Sharing Statement
