# Data Errors in Abstract and Main Results

**DOI:** 10.1001/jamanetworkopen.2025.54094

**Published:** 2025-12-26

In the Original Investigation titled “Access to Prostate-Specific Antigen Testing and Mortality Among Men With Prostate Cancer,”^1^ published June 4, 2024, there were typographical errors in adjusted odds ratios and adjusted hazard ratios in the Abstract and the main Results. These adjusted odds ratios and hazard ratios were reported for changes per 10% increase in county-level prostate-specific antigen testing instead of for quintile 5 vs quintile 1. This error affected the results in the text for associations between testing and staging, all-cause mortality, and prostate cancer-specific mortality. This article has been corrected.^1^
