# Supplementary material for: Access to Prostate-Specific Antigen Testing and Mortality Among Men With Prostate Cancer
Source: JAMA Netw Open. 2024 Jun 4;7(6):e2414582. doi: 10.1001/jamanetworkopen.2024.14582 (PMC11151156; doi:10.1001/jamanetworkopen.2024.14582)
Supplement: Supplement 1. — eMethods. Geospatial Covariates and Time-Varying Exposure eReferences. eFigure 1. CONSORT Diagram for METRO Prostate Cancer Cohort Study of County-Level Prostate-Specific Antigen Testing and Mortality eFigure 2. Kaplan-Meier Survival Curves for County-Level PSA at (A) Baseline, (B) Diagnosis, (C) Cumulative Updated Average in METRO eTable 1. Descriptive Characteristics of the Multilevel Tumor Registry for Evaluation of Oncology (METRO) Cohort of Men With Prostate Cancer Stratified by County-Level Prevalence of Prostate Specific Antigen Testing at Diagnosis eTable 2. Sensitivity Analyses for Associations of County-Level Prevalence of Screening With Advanced Stage, All-Cause Mortality and Prostate Cancer-Specific Mortality Stratified by Race (Prostate-Specific Antigen), and With Mammography as a Negative Control [file jamanetwopen-e2414582-s001.pdf]

Supplementary Online Content

Iyer HS, Stone BV, Roscoe C, et al. Access to prostate-specific antigen testing and mortality among men with prostate cancer. *JAMA Netw Open.* 2024;7(6):e2414582. doi:10.1001/jamanetworkopen.2024.14582

eMethods. Geospatial Covariates and Time-Varying Exposure

eReferences.

eFigure 1. CONSORT Diagram for METRO Prostate Cancer Cohort Study of County-Level Prostate-Specific Antigen Testing and Mortality

eFigure 2. Kaplan-Meier Survival Curves for County-Level PSA at (A) Baseline, (B) Diagnosis, (C) Cumulative Updated Average in METRO

eTable 1. Descriptive Characteristics of the Multilevel Tumor Registry for Evaluation of Oncology (METRO) Cohort of Men With Prostate Cancer Stratified by County-Level Prevalence of Prostate Specific Antigen Testing at Diagnosis

eTable 2. Sensitivity Analyses for Associations of County-Level Prevalence of Screening With Advanced Stage, All-Cause Mortality and Prostate Cancer-Specific Mortality Stratified by Race (Prostate-Specific Antigen), and With Mammography as a Negative Control

This supplementary material has been provided by the authors to give readers additional information about their work.

© 2024 Iyer HS et al. *JAMA Network Open.*

eMethods. Geospatial Covariates and Time-Varying Exposure

Geospatial neighborhood and contextual measures

In order to evaluate potential environmental and spatial access-related factors associated with CaP outcomes, we obtained geomasked residential addresses for participants. We identified neighborhood contextual factors, structural and social determinants of health, and pollutants that have been linked to poorer survival and access in men with prostate cancer[1–5]. For Louisiana, spatial databases were sent to the Louisiana Tumor Registry along with documentation and software to append spatial data to exact residential addresses. For all other states, geomasking was performed using a radial buffer, with residential address geocodes randomly displaced within 250m of the participant's address in urban areas and 400m in rural areas. In addition, the direction of displacement was randomly selected to be north or south (latitude) or east or west (longitude). Further details regarding use of address-level data linkages while balancing privacy concerns and regulations have been described previously.[6]

Structural and Social Determinants of Health

We included neighborhood measures of structural and social determinants of health because these upstream sociopolitical factors can drive disparate outcomes in socially disadvantaged communities[1,7,8]. Structural determinants refer to policies and cultural norms that preferentially benefit some groups and disadvantage others, most commonly applied to racism[8]. Social determinants refer to material resources and hierarchies based on wealth, income, and class that can influence health indirectly through access to care, health behaviors, and environmental exposures[9,10]. We obtained data on racial composition, income, wealth, occupation, and other socioeconomic factors from the Census Tract-level data from the 2000 Decennial Census and the 2006-2010 American Community Survey and appended these to participant's addresses at diagnosis (for participants diagnosed prior to 2005, we linked 2000 measures, for participants diagnosed 2006 or later, we linked 2006-2010 measures).

In order to capture structural determinants of health in our models, we included measures of the Index of Concentration at the Extremes (ICE), which captures "social spatial polarization" and may be a marker of residential segregation by race and class[11]. The ICE is calculated for a given geography and ranges from -1 to 1, with lower values corresponding to neighborhoods with concentrated disadvantage, and higher values corresponding to neighborhoods with concentrated privilege. Axes of disadvantage and privilege include race (Black vs White), income (highest vs lowest quintile of median income), and racialized income (Black lowest quintile of income vs White highest quintile of income). We calculated ICE Race, ICE Income, and racialized income ICE at Census Tract-level and appended to participant's at their year of diagnosis as described above.

We calculated a neighborhood socioeconomic status index using previously described methods[12]. In brief, we obtained a set of previously validated Census Tract-level measures of socioeconomic status (median income, median home value, % college educated, % families receiving interests and dividends, % occupied housing units, % White, % Black, % foreign-born, and % unemployed). We then z-scaled and summed these indicators to generate a simple z-score based nSES index that was then linked to participant residential Census Tracts at their year of diagnosis as described above.

Residential greenness

Residential greenness, or leafy vegetation around one's residence, is associated with lower all-cause mortality[13], risk of lethal prostate cancer in urban settings[3] and lower mortality in men with prostate cancer[2]. We approximated exposure to residential greenness using satellite-derived Normalized Difference

© 2024 Iyer HS et al. *JAMA Network Open.*

Vegetation Index (NDVI) obtained at 30m resolution from Landsat 7 and Landsat 8 satellites for the period from 2000 through 2018. The NDVI ranges from -1 to 1, with values 0 and above indicating increasing levels of green leafy vegetation[14]. Using Google Earth Engine[15], we first calculated focal statistics to spatially smooth across 270m by 270m buffers. We then selected the least cloudy image for each season (January-March, April-June, July-September, October-December) and estimated the annual seasonal average NDVI during the year of diagnosis as previously described[16]. In a multi-state study, using seasonal measures rather than peak (July) NDVI allows us to capture geographic variation in greenness exposure for all participants.

Air pollution

Outdoor air pollution has been classified as a Group 1 (carcinogenic to humans) carcinogen by the International Agency for Research on Cancer (IARC)[17]. Particulate matter within 2.5 microns in diameter ($PM_{2.5}$) is associated with higher lung cancer, poorer cardiovascular health, and higher mortality[18–20]. We obtained annual measures of PM2.5 estimated using ensemble models that combined predictions from neural network, gradient boosting, and random forest methods[21]. Models took as inputs spatiotemporal data from satellites, meteorologic monitoring, land cover, elevation, and chemical transport models. The cross-validated R^2 for the models was 0.89. Further details regarding the approach and validation are available elsewhere[21].

Data were accessed from the Columbia Socioeconomic Data and Applications Center at 1km resolution for the years 2000 through 2016[22]. We assigned predicted outdoor $PM_{2.5}$ measures from the 1km x 1km pixel containing the participant's geomasked residential address at diagnosis. Exposure was modeled as a quadratic term to account for potential non-linear associations with stage at diagnosis and survival.

Travel burden

Prostate cancer patients experiencing longer travel times to facilities often have lower receipt of guideline-concordant treatment[5,23], although some studies find longer travel times are associated with improved survival[24]. Reasons for these discrepancies may relate to reliance on Euclidean or straight-line distances, which do not account for traffic patterns or mode of transport (public transport vs driving). To overcome these limitations, we generated travel time zones around cancer care facilities (<30 minutes, 30-59 minutes, ≥60 minutes) using the Travel Time platform which generates isochrones (polygons) around each facility to represent the area in which travel time for residents to reach that facility are within the threshold. Time-based estimates are estimated from Google Maps' API and only available for 6-12 months. Therefore, we used weekday morning times during fall 2022 to generate zones.

For facility locations, we obtained locations from the 2000 ESRI Business Analyst database and assigned cancer facilities using North American Industry Classification System (NAICS) codes that indicate health care delivery (using keyword "Cancer" and excluding pharmacies).

We then appended the travel time zones to participants based on their geomasked residential address.

Health systems resources

Data on provider density (urologist, radiation oncologists, primary care providers) were obtained from the 2012 Area Health Resource File, an inventory of health systems and utilization monitoring data compiled by the Health Resources & Services Administration in the US Department of Health & Human Services[25]. We obtained county-level data from 2012 and calculated density of providers in counties

© 2024 Iyer HS et al. *JAMA Network Open.*

within states in the METRO. These were spatially appended to participants' geomasked residential address.

Time-varying measures of county-level prostate-specific antigen screening prevalence

Our county-level PSA screening prevalence measures were available for 2004, 2006, 2008, 2010, and 2012. Given that diagnosis years in our study population ranged from 2000 to 2015, we sought to evaluate whether associations of county-level PSA screening prevalence and outcomes varied based on the time period during which county-level PSA screening was assigned. For our primary analysis, we assigned county-level PSA screening prevalence during the two years prior to diagnosis ("diagnosis"). For cases diagnosed in 2004 or earlier, we assigned the county-level screening prevalence for 2004. For cases diagnosed in 2014 or later, we assigned 2012.

We then re-ran analyses using county-level prevalence assessed in 2004 ("baseline"), corresponding to screening during the earliest available year for the cohort. Finally, we estimated a cumulative updated average, taking all the available county-level PSA screening values at the residential address prior to diagnosis (e.g. if a case were diagnosed in 2008, we would take the average of measures in 2004 and 2006). These other exposure windows allowed us to evaluate whether estimates were sensitive to the choice of exposure time window.

Mammography screening for breast cancer

Because we evaluated associations of county-level PSA screening with prostate cancer outcomes, we interpret findings as providing evidence for health systems factors that promote accessibility to PSA screening, which may include other correlated factors (such as quality medical home or referral networks for treatment). To further examine the potential for this, we sought to study a preventive care measure that would be expected to correlate with PSA screening and share similar common causes with all-cause mortality. We selected mammography screening for breast cancer because we would expect similar unmeasured health systems factors (recommendations from primary care providers, referral networks) and patient factors (care seeking behaviors, health care access) to predict prevalence of mammography screening. However, because mammography screening is only performed in women, we would expect attenuation of the association between county-level mammography screening with prostate cancer outcomes. Although shared health systems and patient factors are correlated with mammography screening and prostate cancer outcomes and so some residual association may be observed, we expect attenuation of associations of county-level mammography with our prostate cancer outcomes compared to associations of county-level PSA with prostate cancer outcomes. This method has been referred to as a "negative control"[26].

We obtained data on county-level mammography estimates from the Small Area Estimates for Cancer-Related Measures program, which is part of the Surveillance Research Program in the National Cancer Institute's Division of Cancer Control and Population Sciences[27]. In brief, data were obtained from both the Behavioral Risk Factor Surveillance System and the National Health Interview Survey, the two largest historical health surveys used in the United States to estimate national and state prevalence of different health behaviors and use of preventive services including cancer screening. Models account for sampling (landline, cell phone, and households without phones, as well as non-response). Next, a hierarchical Bayesian model uses direct estimates of county prevalence rates based on phone-based sampling, in which responses for screening and other health behaviors are directly assessed in relation to 26 county-level covariates based on racial and ethnic composition, educational attainment, demographics, income, wealth, and other social environmental factors obtained from the 2000 and 2010 Census and

American Community Survey. Further details regarding the methodology are available on the program website and have been published[27,28].

We assigned county-level prevalence of mammography to prostate cancer cases based on geomasked residential addresses as described in our methods. County-level prevalence of mammography screening was estimated from 1997-1999, 2000-2003, 2004-2007, 2008-2010, 2011-2016, and 2017-2019. To evaluate similar time periods as our PSA screening at diagnosis measure, we assigned mammography screening from 19997-1999 to prostate cancer cases diagnosed from 2000-2003, mammography from 2000-2003 to prostate cancer cases diagnosed from 2004-2007, mammography from 2004-2007 to prostate cancer cases diagnosed from 2008-2010, mammography from 2008-2010 to prostate cancer cases diagnosed from 2010-2012, and mammography from 2011-2016 to all others.

Competing risks analysis

For prostate cancer-specific analysis, we sought to account for competing risks of other causes of death using an extension of inverse probability of censoring weights which are used to analytically control for selection bias[29,30]. In this method, inverse probability weights are used to estimate the probability of remaining in the risk set without censoring due to the competing event, and so provide an estimate of the association between county-level PSA screening with prostate cancer-specific mortality in the absence of the competing event while also reducing bias due to informative censoring.[29] This method also can be implemented in computationally demanding environments with large sample sizes as in this study.

We grouped events as censored, prostate cancer death, or death from other causes, with death from other causes specified as the competing event. We fit logistic regression models to estimate the numerator and denominator of the inverse probability weights, with death from other causes as the outcome. Models for the denominator of the weights were adjusted for age, diagnosis year, race and ethnicity, nSES, racialized income ICE, urologist density (restricted cubic splines with 3 knots), radiation oncologist density (restricted cubic splines with 3 knots), NDVI, $PM_{2.5}$ (restricted cubic splines with 3 knots), travel time, and stage at diagnosis (advanced, regional, distant, and missing). We truncated outliers to the 1st and 99th percentiles and calculated stabilized inverse probability weights using predicted probabilities from the numerator and denominator models[31]. Weights were applied to Cox proportional hazards models, and robust standard errors were specified to account for within individual correlation induced by the weighting procedure for prostate cancer-specific mortality described in the main methods.

Sensitivity analysis

We performed a series of sensitivity analyses to evaluate robustness of findings to different assumptions and potential sources of confounding. We performed a sensitivity analysis using race-specific estimates of county-level PSA screening using methodology described for our main analysis, but weighting separately to Black and White racial and ethnic groups, the two groups for which racial disparities are best characterized. This allowed us to estimate county-specific prevalence of screening the Non-Hispanic Black and Non-Hispanic White populations for the years 2004, 2006, 2008, 2010 and 2012, which were appended to our METRO cohort based on diagnosis year and county of residence as described in our main paper. For this sensitivity analysis, we estimated associations of county-level race-specific screening with advanced stage at diagnosis, all-cause mortality, and prostate cancer-specific mortality, restricting to the population with that racial and ethnic group (e.g. associations of county-level PSA prevalence in Non-Hispanic Black population with prostate cancer outcomes were assessed in the METRO cohort restricted to Non-Hispanic Black individuals). We also repeated main analyses with

county-level mammography in the period prior to diagnosis as described above. Models were adjusted for covariates in (Model 2: Full) set presented in methods.

© 2024 Iyer HS et al. *JAMA Network Open.*

eReferences.

1. Coughlin SS. A review of social determinants of prostate cancer risk, stage, and survival. Prostate Int [Internet]. 2019 Aug 27 [cited 2020 Feb 12]; Available from: http://www.sciencedirect.com/science/article/pii/S2287888219300649

2. Iyer HS, Valeri L, James P, Chen JT, Hart JE, Laden F, et al. The contribution of residential greenness to mortality among men with prostate cancer: a registry-based cohort study of Black and White men. Environ Epidemiol Phila Pa [Internet]. 2020 Apr 9 [cited 2020 Sep 1];4(2). Available from: https://www.ncbi.nlm.nih.gov/pmc/articles/PMC7147390/

3. Iyer HS, James P, Valeri L, Hart JE, Pernar CH, Mucci LA, et al. The association between neighborhood greenness and incidence of lethal prostate cancer: A prospective cohort study. Environ Epidemiol. 2020 Apr;4(2):e091.

4. Parent MÉ, Goldberg MS, Crouse DL, Ross NA, Chen H, Valois MF, et al. Traffic-related air pollution and prostate cancer risk: a case–control study in Montreal, Canada. Occup Environ Med. 2013 Jul 1;70(7):511–8.

5. Muralidhar V, Rose BS, Chen YW, Nezolosky MD, Nguyen PL. Association Between Travel Distance and Choice of Treatment for Prostate Cancer: Does Geography Reduce Patient Choice? Int J Radiat Oncol. 2016 Oct 1;96(2):313–7.

6. Iyer HS, Shi X, Satagopan JM, Cheng I, Roscoe C, McLaughlin RH, et al. Advancing Social and Environmental Research in Cancer Registries Using Geomasking for Address-Level Data. Cancer Epidemiol Biomarkers Prev. 2023 Nov 1;32(11):1485–9.

7. Williams DR, Collins C. Racial Residential Segregation: A Fundamental Cause of Racial Disparities in Health - David R. Williams, Chiquita Collins, 2001. Public Health Rep [Internet]. 2001 Sep 1 [cited 2021 Jan 18]; Available from: http://journals.sagepub.com/doi/abs/10.1093/phr/116.5.404

8. Bailey ZD, Krieger N, Agénor M, Graves J, Linos N, Bassett MT. Structural racism and health inequities in the USA: evidence and interventions. The Lancet. 2017 Apr 8;389(10077):1453–63.

9. Krieger N, Williams DR, Moss NE. Measuring social class in US public health research: concepts, methodologies, and guidelines. Annu Rev Public Health. 1997;18:341–78.

10. Warnecke RB, Oh A, Breen N, Gehlert S, Paskett E, Tucker KL, et al. Approaching Health Disparities From a Population Perspective: The National Institutes of Health Centers for Population Health and Health Disparities. Am J Public Health. 2008 Sep;98(9):1608–15.

11. Krieger N, Waterman PD, Spasojevic J, Li W, Maduro G, Van Wye G. Public Health Monitoring of Privilege and Deprivation With the Index of Concentration at the Extremes. Am J Public Health. 2016 Feb 1;106(2):256–63.

12. DeVille NV, Iyer HS, Holland I, Bhupathiraju SN, Chai B, James P, et al. Neighborhood socioeconomic status and mortality in the nurses' health study (NHS) and the nurses' health study II (NHSII). Environ Epidemiol. 2023 Feb;7(1):e235.

13. Rojas-Rueda D, Nieuwenhuijsen MJ, Gascon M, Perez-Leon D, Mudu P. Green spaces and mortality: a systematic review and meta-analysis of cohort studies. Lancet Planet Health. 2019 Nov 1;3(11):e469–77.

© 2024 Iyer HS et al. *JAMA Network Open.*

14. Kriegler F, Malila W, Nalepka R, Richardson W. Preprocessing transformations and their effects on multispectral recognition. In: Proceedings of the Sixth International Symposium on Remote Sensing of Environment. Ann Arbor, MI: University of Michigan; 1969. p. 97–131.

15. Gorelick N, Hancher M, Dixon M, Ilyushchenko S, Thau D, Moore R. Google Earth Engine: Planetary-scale geospatial analysis for everyone. Remote Sens Environ. 2017 Dec 1;202:18–27.

16. Iyer HS, Hart JE, James P, Elliott EG, DeVille NV, Holmes MD, et al. Impact of neighborhood socioeconomic status, income segregation, and greenness on blood biomarkers of inflammation. Environ Int. 2022 Apr 1;162:107164.

17. Loomis D, Grosse Y, Lauby-Secretan B, Ghissassi FE, Bouvard V, Benbrahim-Tallaa L, et al. The carcinogenicity of outdoor air pollution. Lancet Oncol. 2013 Dec 1;14(13):1262–3.

18. Bhaskaran K, Wilkinson P, Smeeth L. Cardiovascular consequences of air pollution: what are the mechanisms? Heart. 2011 Apr 1;97(7):519–20.

19. Arden Pope Iii C, Burnett RT, Turner MC, Cohen A, Krewski D, Jerrett M, et al. Lung cancer and cardiovascular disease mortality associated with ambient air pollution and cigarette smoke: Shape of the exposure-response relationships. Environ Health Perspect. 2011;119(11):1616–21.

20. Di Q, Wang Y, Zanobetti A, Wang Y, Koutrakis P, Choirat C, et al. Air Pollution and Mortality in the Medicare Population. N Engl J Med. 2017 Jun 29;376(26):2513–22.

21. Di Q, Amini H, Shi L, Kloog I, Silvern R, Kelly J, et al. An ensemble-based model of PM2.5 concentration across the contiguous United States with high spatiotemporal resolution. Environ Int. 2019 Sep 1;130:104909.

22. Di Q, Wei Y, Shtein A, Hultquist C, Xing X, Amini H, et al. Daily and Annual PM2.5 Concentrations for the Contiguous United States, 1-km Grids, v1 (2000 - 2016) [Internet]. Palisades, New York: NASA Socioeconomic Data and Applications Center (SEDAC); Available from: https://doi.org/10.7927/0rvr-4538

23. Mahal BA, Chen YW, Sethi RV, Padilla OA, Yang DD, Chavez J, et al. Travel distance and stereotactic body radiotherapy for localized prostate cancer. Cancer. 2018;124(6):1141–9.

24. Vetterlein MW, Löppenberg B, Karabon P, Dalela D, Jindal T, Sood A, et al. Impact of travel distance to the treatment facility on overall mortality in US patients with prostate cancer. Cancer. 2017;123(17):3241–52.

25. Bureau of Health Professions. User Documentation for the Area Health Resource File (AHRF) 2012-2013 Release. U.S. Department of Health and Human Services; 2013.

26. Lipsitch M, Tchetgen ET, Cohen T. Negative Controls: A Tool for Detecting Confounding and Bias in Observational Studies. Epidemiol Camb Mass. 2010 May;21(3):383–8.

27. Methodology for the Model-based Small Area Estimates of Cancer Risk Factors and Screening Behaviors - Small Area Estimates | SRP/DCCPS/NCI/NIH [Internet]. [cited 2024 Mar 27]. Available from: https://sae.cancer.gov/nhis-brfss/methodology.html

© 2024 Iyer HS et al. *JAMA Network Open.*

28. Liu B, Parsons V, Feuer EJ, Pan Q, Town M, Raghunathan TE, et al. Small Area Estimation of Cancer Risk Factors and Screening Behaviors in US Counties by Combining Two Large National Health Surveys. Prev Chronic Dis. 2019 Aug 29;16:E119.

29. Hernán MA, Hernández-Díaz S, Robins JM. A structural approach to selection bias. Epidemiol Camb Mass. 2004 Sep;15(5):615–25.

30. Matsuyama Y, Yamaguchi T. Estimation of the marginal survival time in the presence of dependent competing risks using inverse probability of censoring weighted (IPCW) methods. Pharm Stat. 2008;7(3):202–14.

31. Cole SR, Hernán MA. Constructing Inverse Probability Weights for Marginal Structural Models. Am J Epidemiol. 2008 Sep 15;168(6):656–64.

© 2024 Iyer HS et al. *JAMA Network Open.*

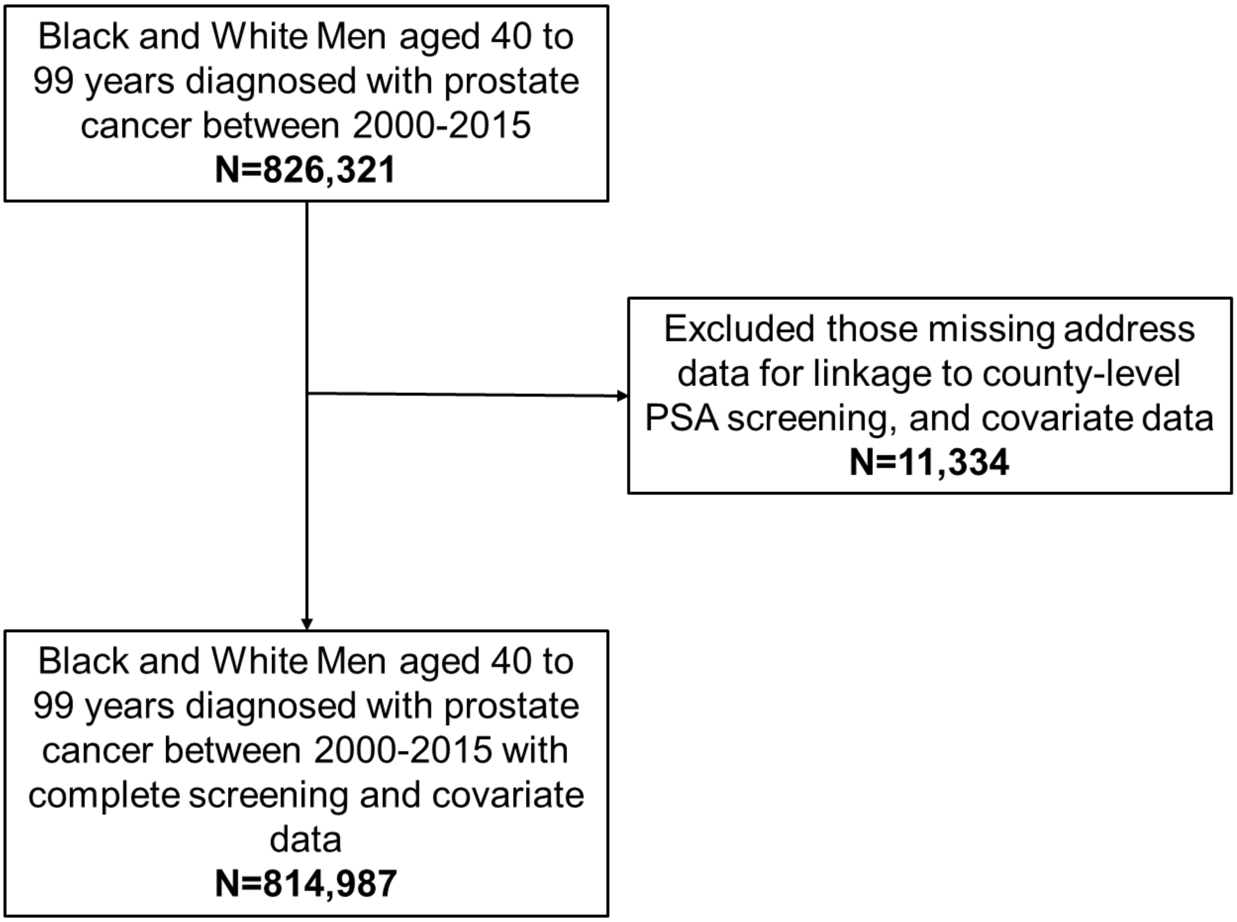

eFigure 1. CONSORT Diagram for METRO Prostate Cancer Cohort Study of County-Level Prostate-Specific Antigen Testing and Mortality

© 2024 Iyer HS et al. *JAMA Network Open.*

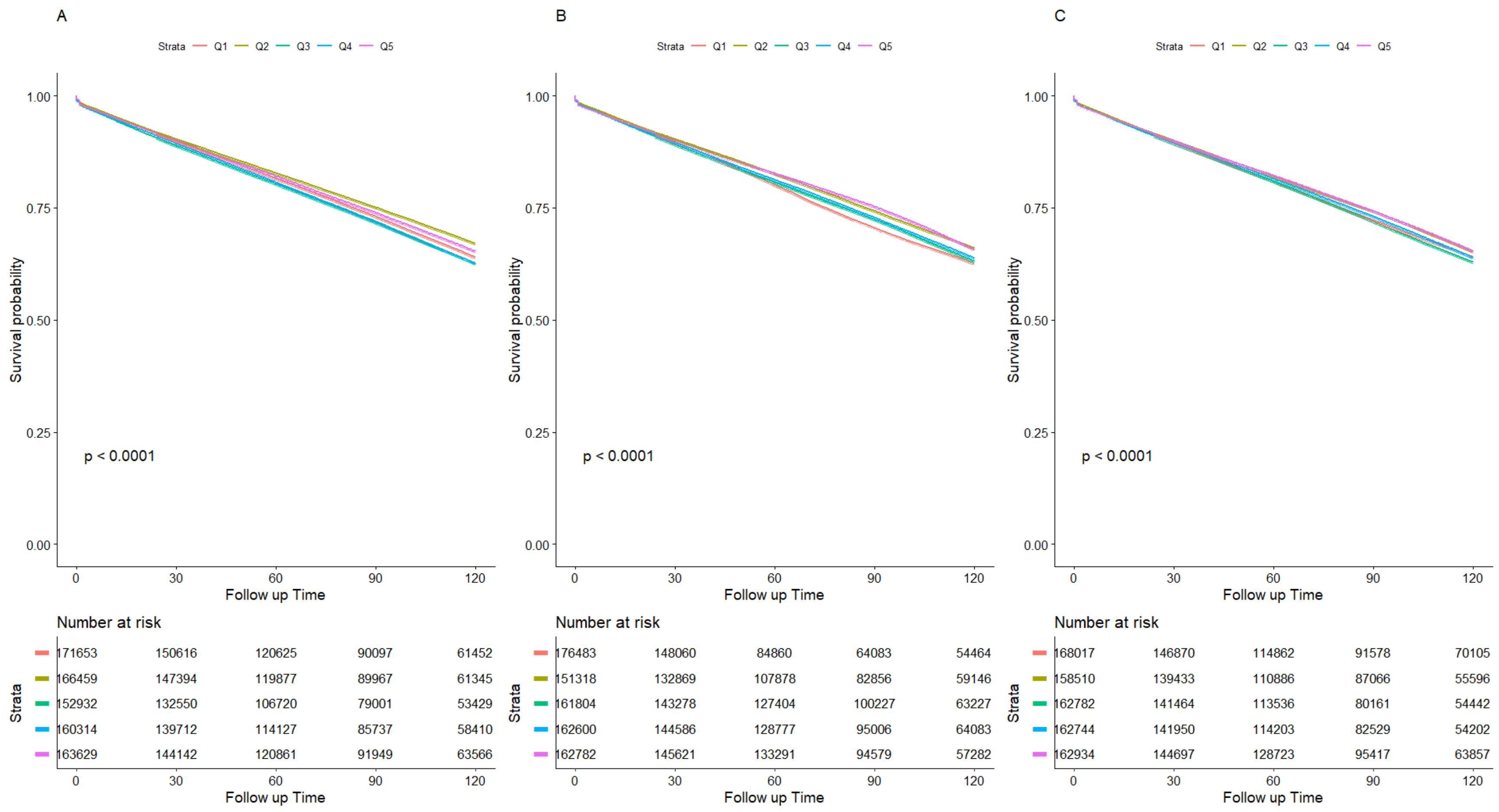

eFigure 2. Kaplan-Meier Survival Curves for County-Level PSA at (A) Baseline, (B) Diagnosis, (C) Cumulative Updated Average in METRO

© 2024 Iyer HS et al. *JAMA Network Open.*

eTable 1. Descriptive Characteristics of the Multilevel Tumor Registry for Evaluation of Oncology (METRO) Cohort of Men With Prostate Cancer Stratified by County-Level Prevalence of Prostate Specific Antigen Testing at Diagnosis

**County-level PSA prevalence at diagnosis**<br>**Range (minimum, maximum)**	**Quintile 1**<br>38.1%-50.2%	**Quintile 2**<br>50.3%-53.6%	**Quintile 3**<br>53.7%-55.8%	**Quintile 4**<br>55.9%-58.5%	**Quintile 5**<br>58.6%-67.1%	**Overall**
**n**	**176483**	**151318**	**161804**	**162600**	**162782**	**814987**
**Age at diagnosis (mean (SD))**	67.22 (9.71)	66.98 (9.76)	67.58 (9.88)	67.53 (9.76)	67.21 (9.73)	67.31 (9.77)
**Race and ethnicity (%)**						
Non-Hispanic White	129513 (73.4)	119372 (78.9)	125177 (77.4)	135613 (83.4)	142197 (87.4)	651872 (80.0)
Non-Hispanic Black	24850 (14.1)	17086 (11.3)	23429 (14.5)	18788 (11.6)	15059 (9.3)	99212 (12.2)
Hispanic	22120 (12.5)	14860 (9.8)	13198 (8.2)	8199 (5.0)	5526 (3.4)	63903 (7.8)
**Insurance (%)**						
Private	46936 (49.8)	48042 (51.4)	43663 (47.1)	52889 (46.6)	53456 (43.2)	244986 (47.3)
Uninsured	4788 (5.1)	4538 (4.9)	6497 (7.0)	5815 (5.1)	8622 (7.0)	30260 (5.8)
Medicaid	5144 (5.5)	3572 (3.8)	3140 (3.4)	2650 (2.3)	2298 (1.9)	16804 (3.2)
Medicare	36756 (39.0)	36468 (39.0)	38137 (41.1)	50328 (44.3)	57664 (46.6)	219353 (42.4)
Other Govt	610 (0.6)	922 (1.0)	1347 (1.5)	1850 (1.6)	1650 (1.3)	6379 (1.2)
**Year of diagnosis (%)**						
2000-2004	58635 (33.2)	49892 (33.0)	61692 (38.1)	57070 (35.1)	45379 (27.9)	272668 (33.5)
2005-2009	28106 (15.9)	55803 (36.9)	65725 (40.6)	62148 (38.2)	61162 (37.6)	272944 (33.5)
2010-2015	89742 (50.9)	45623 (30.2)	34387 (21.3)	43382 (26.7)	56241 (34.5)	269375 (33.1)
**Marital Status (%)**						
Not married	47972 (29.7)	36262 (27.7)	37574 (24.6)	39391 (25.7)	41208 (28.3)	202407 (27.2)
Married	96876 (60.1)	80752 (61.7)	100488 (65.8)	99885 (65.1)	91237 (62.6)	469238 (63.1)
Missing	16473 (10.2)	13775 (10.5)	14695 (9.6)	14202 (9.3)	13365 (9.2)	72510 (9.7)
**nSES (%)**						
Q1 (Low)	22311 (12.6)	23002 (15.2)	36026 (22.3)	42868 (26.4)	37457 (23.0)	161664 (19.8)
Q2	32483 (18.4)	27993 (18.5)	32339 (20.0)	36557 (22.5)	33129 (20.4)	162501 (19.9)
Q3	38404 (21.8)	31445 (20.8)	32557 (20.1)	29994 (18.4)	30840 (18.9)	163240 (20.0)
Q4	39377 (22.3)	33253 (22.0)	31582 (19.5)	27629 (17.0)	31742 (19.5)	163583 (20.1)
Q5 (High)	43908 (24.9)	35625 (23.5)	29300 (18.1)	25552 (15.7)	29614 (18.2)	163999 (20.1)
**ICE Race (%)**						
Q1	46493 (26.3)	32172 (21.3)	37450 (23.1)	27861 (17.1)	19365 (11.9)	163341 (20.0)
Q2	43296 (24.5)	36844 (24.3)	34956 (21.6)	27464 (16.9)	20624 (12.7)	163184 (20.0)
Q3	38255 (21.7)	32319 (21.4)	31825 (19.7)	31379 (19.3)	29889 (18.4)	163667 (20.1)
Q4	30257 (17.1)	26647 (17.6)	29152 (18.0)	34337 (21.1)	42807 (26.3)	163200 (20.0)
Q5	18182 (10.3)	23336 (15.4)	28421 (17.6)	41559 (25.6)	50097 (30.8)	161595 (19.8)
**ICE Income (%)**						
Q1	32241 (18.3)	25979 (17.2)	37714 (23.3)	37756 (23.2)	28195 (17.3)	161885 (19.9)
Q2	33158 (18.8)	27953 (18.5)	33718 (20.8)	36148 (22.2)	31288 (19.2)	162265 (19.9)

© 2024 Iyer HS et al. *JAMA Network Open.*

Q3	35130 (19.9)	30254 (20.0)	31723 (19.6)	31606 (19.4)	34255 (21.0)	162968 (20.0)
Q4	37209 (21.1)	32760 (21.6)	29462 (18.2)	29419 (18.1)	34947 (21.5)	163797 (20.1)
Q5	38745 (22.0)	34372 (22.7)	29187 (18.0)	27671 (17.0)	34097 (20.9)	164072 (20.1)
**ICE Race/Income (%)**						
Q1	40320 (22.8)	28835 (19.1)	38766 (24.0)	32305 (19.9)	22514 (13.8)	162740 (20.0)
Q2	33865 (19.2)	28712 (19.0)	34140 (21.1)	36500 (22.4)	29064 (17.9)	162281 (19.9)
Q3	32862 (18.6)	31026 (20.5)	31299 (19.3)	33007 (20.3)	34437 (21.2)	162631 (20.0)
Q4	34448 (19.5)	32221 (21.3)	29456 (18.2)	31433 (19.3)	35934 (22.1)	163492 (20.1)
Q5	34988 (19.8)	30524 (20.2)	28143 (17.4)	29355 (18.1)	40833 (25.1)	163843 (20.1)
**Stage (%)**						
Localized	115819 (80.2)	97249 (82.5)	91969 (83.5)	96050 (84.6)	83211 (85.3)	484298 (83.0)
Regional	19580 (13.6)	14438 (12.2)	12699 (11.5)	12236 (10.8)	10359 (10.6)	69312 (11.9)
Distant	9066 (6.3)	6186 (5.2)	5521 (5.0)	5195 (4.6)	4019 (4.1)	29987 (5.1)
**State (%)**						
California	84769 (48.0)	67520 (44.6)	57941 (35.8)	45858 (28.2)	25487 (15.7)	281575 (34.5)
Pennsylvania	19150 (10.9)	21719 (14.4)	44516 (27.5)	28820 (17.7)	29294 (18.0)	143499 (17.6)
Ohio	19857 (11.3)	9486 (6.3)	13965 (8.6)	36597 (22.5)	25718 (15.8)	105623 (13.0)
New Jersey	4323 (2.4)	8850 (5.8)	18047 (11.2)	20520 (12.6)	49896 (30.7)	101636 (12.5)
New Mexico	3904 (2.2)	7944 (5.2)	1353 (0.8)	2955 (1.8)	1296 (0.8)	17452 (2.1)
Massachusetts	14019 (7.9)	20003 (13.2)	8719 (5.4)	7488 (4.6)	16202 (10.0)	66431 (8.2)
Louisiana	1011 (0.6)	6159 (4.1)	13373 (8.3)	16926 (10.4)	13721 (8.4)	51190 (6.3)
Seattle/Puget Sound (Washington)	29450 (16.7)	9637 (6.4)	3890 (2.4)	3436 (2.1)	1168 (0.7)	47581 (5.8)

Abbreviations: ICE = index of concentration at the extremes, nSES = neighborhood socioeconomic status, PSA=prostate-specific antigen

© 2024 Iyer HS et al. *JAMA Network Open.*

eTable 2. Sensitivity Analyses for Associations of County-Level Prevalence of Screening With Advanced Stage, All-Cause Mortality and Prostate Cancer-Specific Mortality Stratified by Race (Prostate-Specific Antigen), and With Mammography as a Negative Control

	Continuous (10%)	Quintile 1	Quintile 2	Quintile 3	Quintile 4	Quintile 5	P_{trend}
**Advanced stage**							
County-level PSA at diagnosis[a]	0.86 (0.85, 0.88)	Ref	0.94 (0.92, 0.96)	0.87 (0.85, 0.89)	0.85 (0.83, 0.87)	0.83 (0.81, 0.86)	<.001
Non-Hispanic White[b] (N=459,103)	0.92 (0.91, 0.94)	Ref	0.94 (0.91, 0.96)	0.89 (0.87, 0.92)	0.91 (0.89, 0.94)	0.85 (0.83, 0.88)	<.001
Non-Hispanic Black[b] (N=69,777)	0.91 (0.87, 0.94)	Ref	0.93 (0.89, 0.99)	0.97 (0.91, 1.04)	0.91 (0.85, 0.98)	0.77 (0.71, 0.83)	<.001
County-level mammography at diagnosis[a]	0.89 (0.88, 0.91)	Ref	0.98 (0.96, 1.00)	0.96 (0.94, 0.98)	0.91 (0.89, 0.93)	0.84 (0.82, 0.87)	<.001
**All-cause Mortality**							
County-level PSA at diagnosis[a]	0.86 (0.85, 0.87)	Ref	0.94 (0.93, 0.95)	0.94 (0.93, 0.95)	0.88 (0.86, 0.89)	0.85 (0.84, 0.86)	<.001
Non-Hispanic White[b] (N=651,872)	0.91 (0.90, 0.92)	Ref	0.97 (0.95, 0.98)	0.96 (0.94, 0.98)	0.91 (0.89, 0.92)	0.87 (0.86, 0.88)	<.001
Non-Hispanic Black[b] (N=99,212)	0.97 (0.95, 0.99)	Ref	0.98 (0.95, 1.01)	0.95 (0.92, 0.98)	0.98 (0.94, 1.02)	0.95 (0.91, 1.00)	0.018
County-level Mammography at diagnosis[a]	0.94 (0.93, 0.95)	Ref	1.01 (0.99, 1.02)	1.01 (1.00, 1.03)	0.96 (0.95, 0.98)	0.92 (0.90, 0.93)	<.001
**Prostate Cancer-specific Mortality[c]**							
County-level PSA at diagnosis[a]	0.83 (0.81, 0.85)	Ref	0.94 (0.92, 0.97)	0.93 (0.90, 0.95)	0.85 (0.83, 0.87)	0.82 (0.79, 0.84)	<.001
Non-Hispanic White[b] (N=651,872)	0.89 (0.88, 0.91)	Ref	0.94 (0.91, 0.97)	0.91 (0.89, 0.94)	0.88 (0.85, 0.90)	0.84 (0.81, 0.87)	<.001
Non-Hispanic Black[b] (N=99,212)	0.92 (0.88, 0.96)	Ref	0.89 (0.85, 0.95)	0.86 (0.80, 0.92)	0.90 (0.84, 0.97)	0.90 (0.83, 0.98)	<.001
County-level Mammography at diagnosis[a]	0.93 (0.92, 0.95)	Ref	1.01 (0.99, 1.03)	1.04 (1.01, 1.06)	0.99 (0.96, 1.01)	0.90 (0.87, 0.92)	<.001

[a]Models adjusted for age, diagnosis year, race and ethnicity, nSES, racialized income ICE, greenness, air pollution ($PM_{2.5}$) (quadratic), population density (quadratic), and state

[b]Models adjusted for all covariates in [a] except for race and ethnicity

[c]Models fit using stabilized inverse probability weights for competing event of non-prostate cancer-specific deaths.

© 2024 Iyer HS et al. *JAMA Network Open.*
