# Supplementary material for: Access to Prostate-Specific Antigen Testing and Mortality Among Men With Prostate Cancer
Source: JAMA Netw Open. 2024 Jun 4;7(6):e2414582. doi: 10.1001/jamanetworkopen.2024.14582 (PMC11151156; doi:10.1001/jamanetworkopen.2024.14582)
Supplement: Supplement 2. — Data Sharing Statement [file jamanetwopen-e2414582-s002.pdf]

Data Sharing Statement

Iyer. Access to Prostate-Specific Antigen Testing and Mortality Among Men With Prostate Cancer. *JAMA Netw Open*. Published June 04, 2024.
doi:10.1001/jamanetworkopen.2024.14582

Data
Data available: No

Additional Information
Explanation for why data not available: Data for this study were requested from each registry for the purposes of this research, and Institutional Review Board and Data Use Agreements between Rutgers, Dana-Farber, and each registry prohibit sharing of these data outside of the research team. Inquiries can be directed to: hi97@cinj.rutgers.edu
